# Safety of flexible bronchoscopy and clinical course for elderly patients with suspected primary lung cancer

**DOI:** 10.1111/1759-7714.15228

**Published:** 2024-01-28

**Authors:** Zentaro Saito, Issei Oi, Takanori Ito, Takuma Imakita, Osamu Kanai, Kohei Fujita, Tadashi Mio

**Affiliations:** ^1^ Division of Respiratory Medicine National Hospital Organization Kyoto Medical Center Kyoto Japan

**Keywords:** elderly, flexible bronchoscopy, lung cancer

## Abstract

**Background:**

There have been several reports demonstrating the safety of flexible bronchoscopy (FB) in the elderly, but none have focused specifically on lung cancer, which is a frequent biopsy procedure.

**Methods:**

In this study, we retrospectively evaluated the safety of FB and subsequent treatment in elderly patients with suspected primary lung cancer. Elderly patients were defined as 75 years of age or older.

**Results:**

A total of 141 patients, 77 in the elderly group and 64 in the nonelderly group, were reviewed. The median age of the elderly group was 80 years. Transbronchial lung biopsy was performed in more than 80% of all patients. Primary lung cancer was diagnosed in 42 (54.4%) of the elderly group and 35 (54.7%) of the nonelderly group (*p* = 0.38). Approximately 70% of the elderly patients with a confirmed diagnosis were treated, while more than half of the undiagnosed elderly patients had best supportive care. Complications such as bleeding, pneumothorax, fever, and pneumonia were similar in the elderly and nonelderly groups.

**Conclusions:**

This study suggests that flexible bronchoscopy can be performed as safely in the elderly as in the nonelderly. Furthermore, even elderly patients may have a greater chance of receiving treatment when a definitive diagnosis is achieved.

## Graphical abstract

We examined the safety, diagnosis, and clinical course of flexible bronchoscopy in the elderly. Our study suggests that elderly patients can receive FB as safely as nonelderly patients. Furthermore, even elderly patients may be more likely to receive treatment when a definitive diagnosis is made.

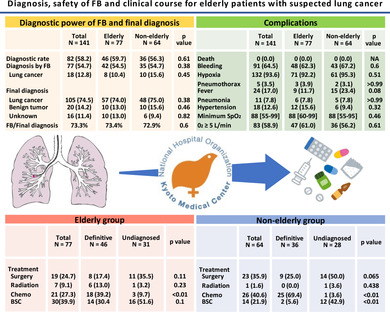

## INTRODUCTION

Cancer is the leading cause of disease burden worldwide, and the global cancer burden is projected to continue growing for at least the next 20 years.^1^, ^2^ Among the 22 groups of diseases in the Global Burden of Diseases (GBD) 2019 study, cancer was the second most common cause of death in 2019. There were 2.04 million deaths and 2.26 million cases of tracheal, bronchial, and lung cancer; these were the most common and leading causes of death in 58 and 119 countries and regions, respectively, in men and one and 27 country and regions, respectively, in women.^3^

Elderly patients with cancer often have comorbidities such as pulmonary and cardiac disease and may be more susceptible to comorbidity‐related adverse events. There have been several reports on flexible bronchoscopy (FB) in elderly patients;^4^, ^5^ however, few reports have focused specifically on lung cancer,^6^ although bronchoscopy methods vary by disease. In lung cancer practice, wide molecular profiling as a biomarker test is recommended to evaluate potential genetic variants, and tissue sampling is crucial.^7^ As a result, the frequency of biopsies for tissue sampling increases when lung cancer is suspected; however, the safety of FB has not been adequately evaluated in elderly patients with suspected lung cancer. Moreover, a previous report demonstrated the efficacy and safety of anticancer therapy in lung cancer patients over 75 years of age.^8^ It is important to determine whether elderly patients receive sufficient treatment after a bronchoscopic diagnosis.

In this study, we investigated the efficacy and safety of FB for suspected primary lung cancer and clinical course in elderly patients.

## METHODS

### Study design and patients

This retrospective study examined the medical records of patients who underwent FB for suspected primary lung cancer at the National Hospital Organization, Kyoto Medical Center, between April 2021 and March 2022. Patients who underwent thoracic drainage or home oxygen therapy prior to FB, or those with incomplete clinical data, were also excluded from this study. The study group consisted of patients aged ≥75 years, and the control group consisted of patients aged 18–74. Bronchoscopy procedure records included examination time, sedation, sampling method, diagnosis, and post‐procedural complications. Indications for FB were categorized by two or more pulmonologists as the presence of nodules/masses, infiltrates, and ground‐glass opacity based on computed tomography (CT) findings. Lesion size was classified into <3 cm, 3–5 cm, and >5 cm according to the T factor of the TNM classification.^9^ The location of the lesion was divided into central and peripheral lesions, with peripheral lesions denied as lesions peripheral to the subregional branches. The study protocol was approved by the Ethics Committee and Institutional Review Board of the National Hospital Organization Kyoto Medical Centre (approval no.: 22‐072). The study adhered to the principles of the World Medical Association Declaration of Helsinki, and the results were reported in accordance with the Strengthening the Reporting of Observational Studies in Epidemiology (STROBE) statement.

### Bronchoscopy procedures

All bronchoscopies were performed on admission to our hospital. Patients were discharged the day after the examination if there were no anticoagulant adjustments or complications associated with FB. Electrocardiograms were monitored only during FB. Bronchoscopy was performed using modified methods recommended by the British Thoracic Society (BTS) and the American College of Chest Physicians (ACCP).^10^, ^11^

Informed consent was obtained from all patients who underwent FB and they fasted for at least 6 h before the procedure. In accordance with the BTS and ACCP guidelines, anticoagulants and P2Y12 receptor antagonists were discontinued before the procedure; however, aspirin was not discontinued. Bronchoscopy was performed using BF‐260, BF‐P290, and BF‐UC290F flexible bronchoscopes (Olympus Corporation). The larynx was anesthetized using 8% lidocaine, and midazolam (1 mg/mL) was used for sedation, which was repeated until sedation was achieved. Opioid analgesics such as fentanyl were not used. The procedure time was defined as the time from insertion of FB to the chest radiograph at the end of the procedure.

The bronchoscope was inserted orally, and sampling methods, such as endobronchial biopsy (EBB), transbronchial biopsy/transbronchial lung biopsy (TBB/TBLB), radial endobronchial ultrasound (EBUS), endobronchial ultrasound‐guided transbronchial needle aspiration (EBUS‐TBNA), and protective specimen brushing, were performed at the discretion of the bronchoscopist. Cryobiopsy and electromagnetic navigation were not performed at our institution; radial endobronchial ultrasound or virtual bronchoscopic navigation was performed at the clinician's discretion. The patient's vital signs, including electrocardiograms, pulse, and blood pressure, were closely monitored throughout the examination. In cases of significant desaturation (saturation of percutaneous oxygen [SpO_2_] <90%), oxygen was administered to maintain the SpO_2_ at 90%. Nicardipine was administered to patients with elevated blood pressure as needed at the discretion of the bronchoscopist. Chest radiographs were obtained at the end of the procedure to confirm the occurrence of pneumothorax, and flumazenil was administered for arousal. All bronchoscopies were performed under the supervision of a specialist physician, and adverse events were observed for ~24 h after FB.

### Efficacy and complications

The diagnostic power and complications of FB were compared between the elderly and nonelderly groups. The diagnostic rate was defined as the histological diagnosis of suspected primary lung cancer. Additionally, we examined the subsequent treatment of elderly patients with a confirmed diagnosis and those who were undiagnosed. To make a final diagnosis, patients undiagnosed by FB received additional examination, including CT‐guided or surgical biopsy. However, the final diagnosis was unknown if the patient did not wish to undergo additional examinations.

Complications were defined as any procedural event occurring during or after FB, regardless of its apparent association with FB. Adverse events were recorded, including hypoxia (SpO_2_ <90% and oxygen supply), elevated blood pressure (use of nicardipine), arrhythmia, bleeding (requiring intratracheal administration of adrenaline or thrombin), fever (axillary body temperature >37.5°C), pneumonia (use of antibiotics), pneumothorax, and mortality. Nicardipine, adrenaline, and thrombin were administered at the discretion of the bronchoscopist. Also, prophylactic antimicrobials were not used in accordance with BTS guidelines.

### Statistical analysis

Continuous variables for background factors and baseline laboratory data are presented as medians and interquartile ranges. The Mann–Whitney U test was used for group comparisons of continuous variables, and the chi‐square or Fisher's exact tests were used for group comparisons of categorical variables, as appropriate. All statistical analyses were performed using R version 4.0.3 (R Foundation for Statistical Computing, Vienna, Austria). A *p*‐value <0.05 was considered statistically significant.

## RESULTS

**FIGURE 1:**
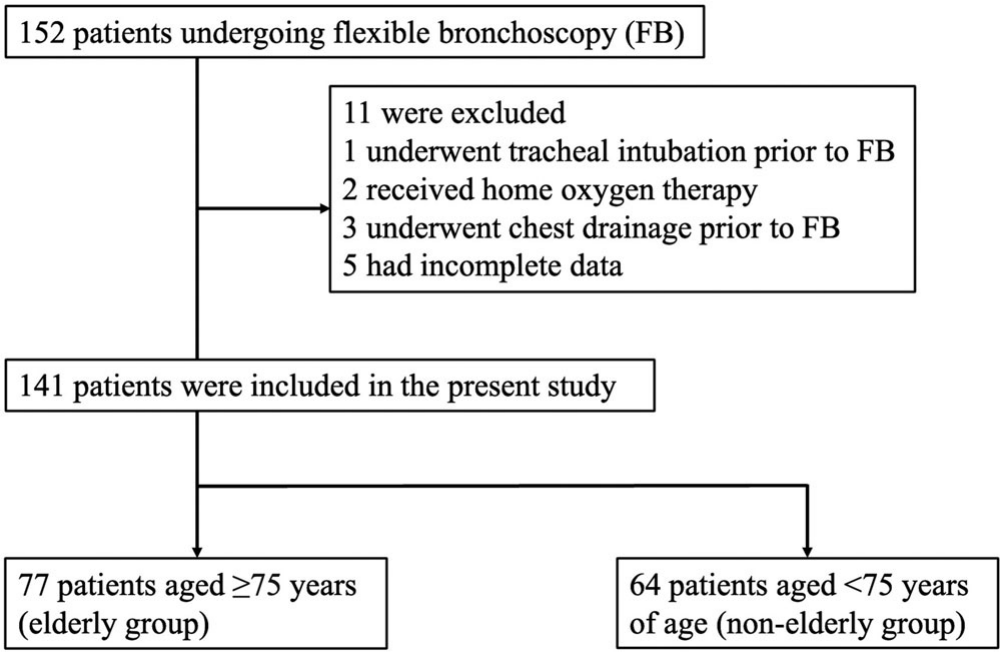
Study flow chart: Screening, patient selection, exclusion, and classification.

Between April 2021 and March 2022, 152 patients suspected of having primary lung cancer underwent FB. Among them, one, two, and three patients who underwent tracheal intubation, home oxygen therapy, and chest drainage, respectively, prior to bronchoscopy were excluded. Additionally, five patients with incomplete data were excluded. Finally, we analyzed the data from 141 patients (Figure 1).

### Patient characteristics

**FIGURE 2:**
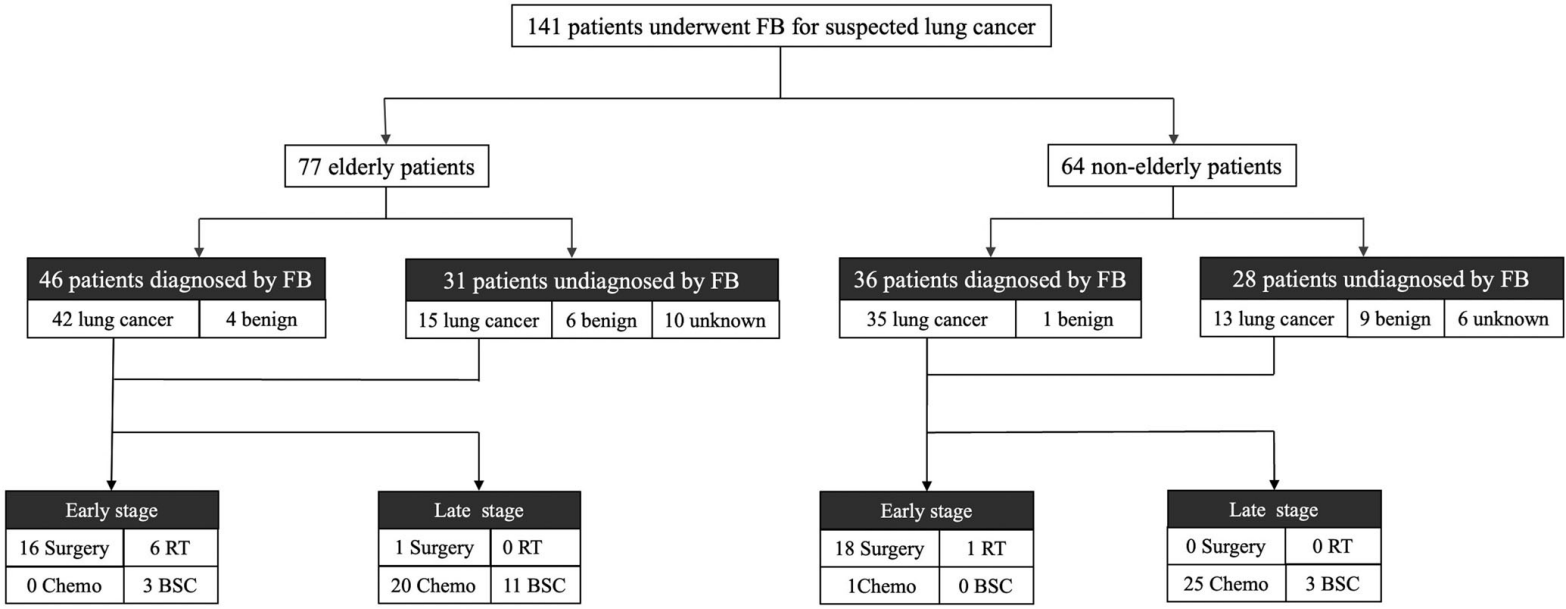
Diagnostic flow chart. Diagnosis by flexible bronchoscopy (FB), final diagnosis, and clinical course. Patients undiagnosed by FB underwent additional examinations, including computed tomography (CT)‐guided or surgical biopsy, in order to reach a final diagnosis. The final diagnosis was unknown if the patient did not consent to undergo additional examinations. BSC, best supportive care; chemo, chemotherapy; RT, radiotherapy.

Patient characteristics, clinical indications, and sampling techniques for flexible bronchoscopy are presented in Table 1. The elderly and nonelderly groups comprised 77 and 64 patients, respectively. The median ages of the elderly and nonelderly groups were 80 and 68 years, respectively. Hypertension was more common in the elderly group than in the nonelderly group (46 [59.7%] vs. 27 [42.2%], respectively, *p* = 0.04), whereas the presence of other comorbidities did not differ between the two groups. The median Charlson comorbidity index was six points in the elderly group and four points in the nonelderly group; the difference was statistically significant. The numbers of patients taking anticoagulants and aspirin were similar in both groups. Blood tests showed no differences in platelet, creatinine, coagulation, or tumor marker levels between the two groups. The pretest vital signs showed a lower pulse rate in the elderly group (75 bpm vs. 81 bpm, *p* = 0.02) (Figure 2).

**TABLE 1:** Patient characteristics and clinical indications, sampling techniques for flexible bronchoscopy.

	Total	Elderly	Nonelderly	*p*‐value
	*N* = 141	*N* = 77	*N* = 64	
Age (years)	75 [33–91]	80 [75–91]	68 [33–74]	NA
Sex (male)	98 (69.5)	50 (64.9)	48 (75.0)	0.2
BMI (kg/m^2^)	21.8 [13.6–30.2]	21.5 [13.6–30.2]	22.3 [15.6–28.4]	0.21
Smoking				
Never (%)	33 (23.4)	28 (36.4)	5 (7.8)	<0.01
ECOG‐PS 0–2	126 (89.4)	68 (88.3)	58 (90.6)	0.79
Anticoagulant	13 (9.2)	10 (13.0)	3 (4.7)	0.14
Aspirin	21 (14.9)	13 (16.9)	8 (12.5)	0.49
Comorbidity				
Hypertension	73 (51.7)	46 (59.7)	27 (42.2)	0.04
Cardiovascular	24 (17.0)	16 (20.8)	8 (12.5)	0.26
Asthma	11 (7.8)	8 (10.4)	3 (4.7)	0.35
COPD	32 (22.7)	16 (20.8)	16 (25.0)	0.69
IP	9 (6.4)	5 (6.5)	4 (6.2)	>0.99
DM	35 (24.8)	22 (28.6)	13 (20.3)	0.33
Cerebrovascular	9 (6.4)	4 (5.2)	5 (7.8)	0.73
Charlson comorbidity index	5.0 [0.0–10.0]	6.0 [4.0–10.0]	4.0 [0.0–8.0]	<0.01
Blood sample data				
Platelet (×10^4^/μL)	25.1 [0.50–66.8]	24.2 [11.4–53.6]	26.6 [0.50–66.8]	0.28
Creatinine (mg/dL)	0.83 [0.39–10.90]	0.88 [0.41–10.90]	0.81 [0.39–9.49]	0.144
PT‐INR	0.93 [0.77–1.7]	0.94 [0.77–1.7]	0.92 [0.77–1.1]	0.35
APTT (sec)	27.4 [20.4–44.3]	27.9 [20.4–40.6]	27.1 [22.4–44.3]	0.11
CEA	3.5 [0.80–1715]	4.10 [0.80–120]	3.25 [0.80–1715]	0.14
SCC	1.3 [0.30–72.5]	1.30 [0.50–72.5]	1.30 [0.30–14.3]	0.49
ProGRP	46.8 [18.4–128 000]	48.45 [20.2–2270]	43.2 [18.4–128 000]	0.16
Vital parameter				
SBP (mmHg)	128 [82–183]	133 [89–170]	124 [82–183]	0.1
DBP (mmHg)	73 [44–110]	73 [44–97]	74 [47–110]	0.4
HR (bpm)	76 [43–122]	75 [43–107]	81 [50–122]	0.02
SpO_2_ (%)	97 [88–100]	98 [88–100]	97 [94–100]	0.56
Indication				
Mass or nodule	120 (85.1)	64 (83.1)	56 (87.5)	0.49
GGO	12 (8.5)	7 (9.1)	5 (7.8)	>0.99
Infiltrate	9 (6.4)	6 (7.8)	3 (4.7)	0.51
Size				
<3 cm	81 (57.4)	48 (62.3)	33 (51.6)	0.23
3–5 cm	42 (29.8)	22 (28.6)	20 (31.2)	0.85
>5 cm	18 (12.8)	7 (9.1)	11 (17.2)	0.21
Lesion				
Central	33 (23.4)	15 (19.5)	18 (28.1)	0.24
Peripheral	108 (76.6)	62 (80.5)	46 (71.9)	0.24
Sampling methods				
TBB/TBLB	113 (80.1)	63 (81.8)	50 (78.1)	0.67
EBB	23 (16.3)	11 (14.3)	12 (18.8)	0.5
EBUS‐TBNA	31 (22.0)	19 (24.7)	12 (18.8)	0.42
Brush	131 (92.9)	70 (90.9)	61 (95.3)	0.35
Radial EBUS	33 (23.4)	18 (23.4)	15 (23.4)	>0.99
Midazolam (mg)	4 [2–10]	4 [3–9]	5 [2–10]	<0.01
Time (min)	20 [6–54]	21 [6–50]	19 [8–54]	0.63

*Note*: Data are shown with median and [range] or number and (percentage).

Abbreviations: APTT, activated partial thromboplastin time; BMI, body mass index; COPD, chronic obstructive pulmonary disease; CEA, carcinoembryonic antigen; DM, diabetes mellitus; DBP, diastolic blood pressure; ECOG‐PS, Eastern Cooperative Oncology Group performance status; EBB, endobronchial biopsy; EBUS‐TBNA, endobronchial ultrasound‐guided transbronchial needle aspiration; GGO, ground‐glass opacity; HR, heart rate; IP, interstitial pneumonia; PT‐INR, prothrombin time‐international normalized ratio; Pro GRP, progastrin‐releasing peptide; SCC, squamous cell carcinoma; SBP, systolic blood pressure; SpO_2_, saturation of percutaneous oxygen; TBB, transbronchial biopsy; TBLB, transbronchial lung biopsy.

The most frequent clinical indication for FB was the presence of masses/nodules. Lesions <3 cm accounted for more than half in total. The nonelderly group had more central lesions, but the difference was not significant. TBB/TBLB was performed in >80% of the cases, with no significant differences between the two groups. The frequency of radial EBUS was ~20% in both groups. The median midazolam dose was lower in the older cohort (4 mg vs. 5 mg, *p* < 0.01). The procedure duration was 20 min in both groups (*p* = 0.63).

### Diagnostic powers of FB and treatments in the elderly and nonelderly groups

Table 2 shows the diagnostic power of FB and final diagnosis in elderly and nonelderly patients. The rate of diagnosis confirmed by FB was similar between the two groups (46 [59.7%] vs. 36 [56.3%], *p* = 0.61). Primary lung cancer was diagnosed in 42 (54.4%) and 35 (54.7%) patients in the elderly and nonelderly groups, respectively (*p* = 0.38). More than half of the patients had stage IV disease, with similar numbers in both groups. Finally, 57 (74.0%) of the elderly group and 48 (75.0%) of the nonelderly group were diagnosed with lung cancer. The FB diagnosis rate of lung cancer relative to the final diagnosis was 73.4% in the elderly group and 72.9% in the nonelderly group. Approximately 70% of the patients with a confirmed diagnosis received treatment. In the elderly group, eight patients each received immune checkpoint inhibitor monotherapy and cytotoxic anticancer therapy.

**TABLE 2:** Diagnostic power of FB and final diagnosis in elderly and nonelderly patients.

	Total	Elderly	Nonelderly	*p*‐value
	*N* = 141	*N* = 77	*N* = 64	
Diagnostic rate	82 (58.2)	46 (59.7)	36 (56.3)	0.61
Diagnosis				
Lung cancer	77 (54.7)	42 (54.5)	35 (54.7)	0.38
Squamous cell carcinoma	18 (12.8)	8 (10.4)	10 (15.6)	0.45
Adenocarcinoma	27 (19.1)	17 (22.1)	10 (15.6)	0.39
Small cell carcinoma	15 (10.6)	8 (10.4)	7 (10.9)	>0.99
NOS	17 (12.1)	9 (11.7)	8 (12.5)	>0.99
Others (including benign tumors)	5 (3.5)	4 (5.2)	1 (1.6)	0.38
Final diagnosis				
Lung cancer	105 (74.5)	57 (74.0)	48 (75.0)	0.46
Benign tumor	20 (14.2)	10 (13.0)	10 (15.6)	0.82
Unknown	16 (11.4)	10 (13.0)	6 (9.4)	0.6
Diagnostic rate FB/final diagnosis	73.3%	73.4%	72.9%	
Stage				
Stage I	34 (24.1)	18 (23.4)	16 (25.0)	0.85
Stage II	11 (7.8)	7 (9.1)	4 (6.3)	0.75
Stage III	11 (7.8)	5 (6.5)	6 (9.4)	0.55
Stage IV	49 (34.7)	27 (35.1)	22 (34.4)	>0.99

*Note*: Data are shown with median and [range] or number and (percentage).

Abbreviation: FB, flexible bronchoscopy; NOS, not otherwise specified.

In contrast, over half of the patients without a diagnosis received best supportive care (BSC). Of the three patients who remained undiagnosed by flexible bronchoscopy and received chemotherapy, two were diagnosed with CT‐guided biopsy, and one was diagnosed clinically with lung cancer. Of the 11 patients who underwent surgery after flexible bronchoscopy, nine were diagnosed with lung cancer on postoperative pathology (Table 3).

**TABLE 3:** Comparison of treatment with and without diagnosis by FB in elderly group.

	Total	Definitive	Undiagnosed	*p*‐value
	*N* = 77	*N* = 46	*N* = 31	
Treatment				
Surgery	19 (24.7)	8 (17.4)	11 (35.5)	0.11
Radiation	7 (9.1)	6 (13.0)	1 (3.2)	0.23
Chemotherapy	21 (27.3)	18 (39.2)	3 (9.7)	<0.01
TKI	5 (6.5)	5 (10.9)	0 (0.0)	
ICI	8 (10.4)	6 (13.0)	2 (6.4)	
Cytotoxic chemo ± ICI	8 (10.4)	7 (15.2)	1 (3.2)	
BSC	30 (39.9)	14 (30.4)	16 (51.6)	0.1

*Note*: Data are shown with number and (percentage).

Abbreviations: BSC, best supportive care; chemo, chemotherapy; FB, flexible bronchoscopy; ICI, immune checkpoint inhibitor; TKI, tyrosine kinase inhibitor.

About 95% of nonelderly patients with a confirmed diagnosis received treatment. Half of the patients without a diagnosis by FB underwent surgery. Two and 21 patients in the nonelderly group received immune checkpoint inhibitor monotherapy and cytotoxic anticancer therapy, respectively. Approximately 40% of undiagnosed patients received BSC (Table 4).

**TABLE 4:** Comparison of treatment with and without diagnosis by FB in the nonelderly group.

	Total	Definitive	Undiagnosed	*p*‐value
	*N* = 64	*N* = 36	*N* = 28	
Treatment				
Surgery	23 (35.9)	9 (25.0)	14 (50.0)	0.065
Radiation	1 (1.6)	0 (0.0)	1 (3.6)	0.438
Chemotherapy	26 (40.6)	25 (69.4)	1 (3.6)	<0.01
TKI	3 (4.7)	3 (8.4)	0 (0.0)	
ICI	2 (3.1)	2 (5.6)	0 (0.0)	
Cytotoxic chemo ± ICI	21 (32.8)	20 (55.6)	1 (3.6)	
BSC	14 (21.9)	2 (5.6)	12 (42.9)	<0.01

*Note*: Data are shown with number and (percentage).

Abbreviations: BSC, best supportive care; chemo, chemotherapy; FB, flexible bronchoscopy; ICI, immune checkpoint inhibitor; TKI, tyrosine kinase inhibitor.

### Complications and vital parameters of FB

Table 5 shows the complications and vital parameters of FB. No patients who underwent FB died during the study period. Bleeding occurred in 48 (62.3%) and 43 (67.2%) patients in the elderly and nonelderly groups, respectively (*p* = 0.6). One (1.6%) and five (6.5%) patients in the nonelderly and elderly groups (*p* = 0.22) required intratracheal administration of thrombin, respectively. Pneumothorax occurred in three patients (3.9%) in the elderly group and two patients (3.1%) in the nonelderly group (*p* > 0.99). Among them, two patients in the elderly group and one in the nonelderly group required thoracic drainage. All patients required prolonged hospitalization; however, their condition improved with thoracic drainage, and surgical intervention was avoided. More than 90% of the patients in both groups required oxygenation, and a maximum oxygen flow of 5 L/min or more was required in ~60% of all patients. Fever was present in 24 (17.0%) patients, with no significant difference between the two groups (9 [11.7%] vs. 15 [23.4%]). Among them, 11 (7.8%) developed pulmonary infections and were treated with antimicrobials, which was similar between the two groups.

**TABLE 5:** Complications and vital parameters by flexible bronchoscopy.

	Total	Elderly	Nonelderly	*p*‐value
	*N* = 141	*N* = 77	*N* = 64	
Death	0 (0.0)	0 (0.0)	0 (0.0)	NA
Bleeding	91 (64.5)	48 (62.3)	43 (67.2)	0.6
Adrenaline	91 (64.5)	48 (62.3)	43 (67.2)	0.6
Thrombin	6 (4.3)	5 (6.5)	1 (1.6)	0.22
Hypoxia	132 (93.6)	71 (92.2)	61 (95.3)	0.51
Pneumothorax	5 (3.5)	3 (3.9)	2 (3.1)	>0.99
Fever	24 (17.0)	9 (11.7)	15 (23.4)	0.08
Pulmonary infection	11 (7.8)	6 (7.8)	5 (7.8)	>0.99
Hypertension	18 (12.6)	12 (15.6)	6 (9.4)	0.32
Arrhythmia	18 (12.6)	12 (15.6)	6 (9.4)	0.32
Vital parameters during FB				
Minimum SpO_2_	88 [55–99]	88 [60–99]	88 [55–95]	0.46
Maximum O_2_ supply (L/min)	5 [0–15]	5 [0–15]	5 [0–15]	0.15
O_2_ supply ≥5 L/min	83 (58.9)	47 (61.0)	36 (56.2)	0.61
Vital parameters after FB				
SBP (mmHg)	132 [86–210]	133 [94–182]	127 [86–210]	0.1
DBP (mmHg)	74 [38–138]	73 [49–117]	74 [38–138]	0.29
HR (bpm)	82 [51–148]	81 [52–116]	83.5 [51–148]	0.32
SpO_2_ (%)	97 [89–100]	97 [89–100]	98 [90–100]	0.46

*Note*: Data are shown with median and [range] or number and (percentage).

Abbreviations: DBP, diastolic blood pressure; FB, flexible bronchoscopy; HR, heart rate; SBP, systolic blood pressure; SpO_2_, percutaneous oxygen saturation.

## DISCUSSION

The present study examined the diagnostic power and complications of FB and clinical course in elderly patients with suspected primary lung cancer. The results showed no significant differences between the elderly and nonelderly groups, demonstrating that FB is effective and safe for elderly patients. This rare study investigated the safety, diagnosis of FB, and clinical course in elderly patients with suspected lung cancer.

The elderly were defined as those aged ≥75 years in this study. No consensus has been achieved on the definition of elderly. However, previous reports of chemotherapy in elderly patients with advanced NSCLC included patients over 75 years of age, and their safety was reported.^8^, ^12^, ^13^ The number of cancer cases among the elderly is increasing, with more than one‐third of new lung cancer cases diagnosed at age 75 or older.^14^ Therefore, we considered it appropriate to define those aged ≥75 years as elderly.

Several reports have examined the safety and efficacy of FB in the elderly, with some defining the elderly as those aged 80 or 85 years. Although the age cutoff in those studies was higher than in the present study, we consider 75 years to be the age criterion for lung cancer treatment.^5^, ^15^, ^16^ However, very few reports have been limited to primary lung cancer. Mineshita et al. reported FB in elderly lung cancer patients, and we consider that the population studied was different from our study. While this was a crucial study on FB in very elderly patients, we used 75 years as the cutoff age and compared them with the nonelderly group. In our study, the elderly group had higher scores on the Charlson comorbidity index. However, there was no difference in FB complications, suggesting that FB could be performed more safely. Moreover, most studies did not mention the bronchoscopy technique or had fewer biopsies, making accurate evaluation difficult. In our report, >80% of all patients experienced TBB/TBLB, and we found no difference in the diagnosis rates or complications between elderly and nonelderly patients.

It is worth noting that in this study, ~70% of the elderly patients received treatment once a definitive diagnosis was made. In contrast, more than half of the undiagnosed elderly patients did not undergo treatment. Also, when compared to the nonelderly group, the elderly group was less likely to receive cytotoxic anticancer therapy and more likely to receive ICI monotherapy. This may reflect the tolerability of anticancer drug treatment for the elderly. The current lung cancer treatment strategy recommends testing for gene variants and programmed cell death ligand 1 immunohistochemistry (PD‐L1 IHC) for patients with metastatic non‐small cell lung cancer.^7^ There are several reports that molecularly targeted agents and immunotherapy could be safely administered to elderly lung cancer patients,^17^, ^18^, ^19^ emphasizing the crucial importance of making a diagnosis by FB, even for elderly patients, in order to receive appropriate treatment.

The frequency of complications in the elderly group was similar to that in the nonelderly group. Although previous studies reported that older age and TBLB are risk factors for bleeding,^20^ our study suggests that TBLB may be performed as safely as in nonelderly patients if FB is performed appropriately. A few cases of pneumothorax drainage were observed; however, surgery could be avoided and did not influence subsequent treatment decisions.

This study had several limitations. First, it was conducted at a single institution. Bronchoscopy procedures varied among hospitals. Since the frequency of complications did not deviate from previous reports, we believe our bronchoscopy method is standard. A multicenter study is required to confirm these findings. Second, it was a retrospective study and individual physicians decided whether to perform bronchoscopy. This may have led to selection bias, and there is a possibility that patients who underwent bronchoscopy had fewer comorbidities. However, there was no difference in the proportion of patients with advanced‐stage disease and Eastern Cooperative Oncology Group performance score 3–4, and we believe that the selection bias was minimal. Third, radial EBUS was used less frequently in our study. However, both groups had similar lesion size and frequency of the peripheral lesions. In this respect, if the use of radial EBUS increases in both groups, the diagnostic rate would be expected to increase to a similar degree. Therefore, we do not believe that this influenced the results.

In conclusion, the present study found that the use of FB elderly patients with suspected primary lung cancer, as well as nonelderly patients, had diagnostic power without severe complications. It was also shown that even at an advanced age, once a definite diagnosis is made, it is linked to appropriate treatment.

## AUTHOR CONTRIBUTIONS

Zentaro Saito: Conceptualization, methodology, formal analysis, writing—original draft, reviewing and editing, and projection administration. Issei Oi: Conceptualization, methodology, reviewing and editing, and projection administration. Takanori Ito, Takuma Imakita, Osamu Kanai, and Kohei Fujita: Writing—review and editing and investigation. Tadashi Mio: Funding acquisition and supervision.

## CONFLICT OF INTEREST STATEMENT

The authors declare that there are no conflicts of interest.
